# Comprehensive genomic and functional analysis of *Leuconostoc* lactic acid bacteria in alcohol and acetaldehyde metabolism

**DOI:** 10.71150/jm.2410026

**Published:** 2025-02-27

**Authors:** Joo-Han Gwak, Yun Ji Choi, Hina Ayub, Min Kyeong Seol, Hongik Kim, Man-Young Jung

**Affiliations:** 1 Department of Biological Sciences and Biotechnology, Chungbuk National University, Cheongju 28644, Republic of Korea; 2 Interdisciplinary Graduate Program in Advance Convergence Technology and Science, Jeju National University, Jeju 63243, Republic of Korea; 3 Vitabio Inc., Sejong 30141, Republic of Korea; 4 Department of Biology Education, Jeju National University, Jeju 63243, Republic of Korea

**Keywords:** *Leuconostoc suionicum* VITA-PB2, lactic acid bacteria (LAB), alcohol and acetaldehyde metabolism, alcohol dehydrogenase (ADH), acetaldehyde dehydrogenase (ALDH)

## Abstract

Alcohol consumption can lead to the accumulation of harmful metabolites, such as acetaldehyde, contributing to various adverse health effects, including hangovers and liver damage. This study presents a comprehensive genomic and functional analysis of *Leuconostoc suionicum* VITA-PB2, a lactic acid bacterial strain isolated from kimchi, to elucidate its role in enhancing alcohol and acetaldehyde metabolism. Genomic characterization revealed key genes encoding alcohol dehydrogenase (ADH) and aldehyde dehydrogenase (ALDH), providing insights into the metabolic capabilities of strain VITA-PB2. Phylogenomic analyses confirmed its taxonomic classification and genetic similarity to other *Leuconostoc* species. Functional validation through in vitro and in vivo experiments demonstrated superior ethanol and acetaldehyde decomposition abilities of strain VITA-PB2, with significant reductions in blood ethanol and acetaldehyde levels observed in rats administered with the strain. Further analysis indicated that while hepatic ADH activity did not significantly increase; however, ALDH expression was elevated. This suggests that the microbial ADH of strain VITA-PB2 contributed to ethanol breakdown, while both microbial and host ALDH facilitated acetaldehyde detoxification. These findings highlight the potential of strain VITA-PB2 as a functional probiotic for mitigating the toxic effects of alcohol consumption.

## Introduction

Alcohol consumption remains a pervasive aspect of many cultures worldwide, but its metabolism in the human body poses substantial health risks. Upon ingestion, ethanol is primarily metabolized in the liver, where it undergoes oxidation by alcohol dehydrogenase (ADH) to form acetaldehyde—a highly reactive and toxic intermediate (Teschke, 2018). Acetaldehyde is a major contributor to the adverse effects associated with alcohol consumption, including symptoms of a hangover such as nausea, vomiting, flushing, and headaches (Erilsson, 2001). Furthermore, chronic exposure to acetaldehyde is linked to more severe conditions, such as liver cirrhosis, cardiovascular diseases, and even cancer (Penning et al., 2010; Seitz et al., 2010).

To mitigate these toxic effects, acetaldehyde is further oxidized to a less harmful and easily excreted metabolite, such as acetate via acetyl coenzyme A (acetyl-CoA) by acetaldehyde dehydrogenase (ALDH), a crucial step in detoxifying alcohol (Chang et al., 2017; Tsermpini et al., 2022) However, the efficiency of this detoxification process can vary significantly among individuals, often leading to the accumulation of acetaldehyde in the bloodstream and contributing to the severity of hangover symptoms (Erilsson, 2001; Mackus et al., 2020). Acetaldehyde, a highly reactive compound, is known to form covalent bonds with proteins, impairing DNA repair mechanisms and increasing carcinogenesis potential (Tuma et al., 1987). This interaction leads to oxidative stress and liver damage, including conditions like fatty liver and hepatic necrosis. Consequently, there is increasing interest in developing functional foods and supplements that enhance the activity of ADH and ALDH, promoting efficient alcohol metabolism and mitigating the toxic effects of alcohol consumption (Moslemi et al., 2022).

Probiotics, particularly lactic acid bacteria (LAB), have emerged as a promising approach in this regard. LAB are well-known for their beneficial effects on gut health, but recent studies have expanded their potential applications to include the enhancement of alcohol metabolism (Jung et al., 2021). Certain LAB strains, including those from the *Leuconostoc* genus, have demonstrated the ability to modulate alcohol metabolism by increasing the activity of ADH and ALDH (Yun et al., 2024). This effect not only accelerates the conversion of ethanol to less harmful metabolites but also reduces the concentration of acetaldehyde in the bloodstream, potentially alleviating the symptoms of alcohol-induced hangovers (Jung et al., 2023).

The efficacy of LAB in promoting alcohol metabolism has been observed in various forms, including both live and heat-killed preparations (Jung et al., 2021, 2023). For instance, studies have shown that *Limosilactobacillus fermentum* (formally *Lactobacillus fermentum*) can significantly decrease blood alcohol levels and enhance liver function by boosting ADH and ALDH activities (Jung et al., 2023). Furthermore, the use of heat-treated LAB has been found to retain these beneficial effects, suggesting that the active components responsible for this enhancement are not compromised by thermal processing (Jung et al., 2021). Given these findings, there is considerable interest in exploring other LAB strains, such as those within the *Leuconostoc* genus, for their potential to improve alcohol metabolism and reduce the adverse effects of alcohol consumption.

This study aims to investigate the impact of feeding a LAB strain, *Leuconostoc suionicum* VITA-PB2, on the degradation of alcohol and acetaldehyde in an in vitro cell system and an in vivo mouse model. Specifically, we assessed the effects of this probiotic on blood ethanol and acetaldehyde concentrations, as well as the activity of ADH and ALDH in liver tissues. A comprehensive genomic analysis was conducted to identify key genes encoding ADH and ALDH, providing fundamental insights into the metabolic capabilities of the strain. The presence of these genes highlights the mechanistic potential of VITA-PB2 to actively degrade ethanol and detoxify acetaldehyde. By elucidating the mechanisms through which *Leuconostoc* species influence alcohol metabolism, this research aims to establish VITA-PB2 as a promising functional ingredient in hangover relief products and other applications targeting the toxic effects of alcohol.

## Materials and Methods

### Preparation and isolation of the LAB strain

The kimchi sample used in this study was a traditionally fermented Korean kimchi, prepared by a local household in Daejeon, Republic of Korea, using conventional ingredients and fermentation methods. This sample was transported under 4ºC and processed for strain isolation immediately upon arrival at the laboratory. The supernatant of the kimchi sample was serially diluted in de Man Rogosa-Sharpe broth medium (MRS; Becton, Dickinson and Company, USA). Thereafter, aliquots of each serial dilution were spread on MRS agar plates, which were then incubated at 37ºC for 2 days. Colonies with distinct morphologies were picked and further purified by repeated streaking on MRS agar. The isolates were preliminarily screened based on their alcohol tolerance in vitro first. The isolates were identified through 16S rRNA gene sequencing following established protocols (Clarridge III, 2004; Johnson et al., 2019). Among the isolates, *Leuconostoc suionicum* VITA-PB2 and *Pediococcus pentosaceus* VITA-PK1 were selected for further study based on their superior ethanol and acetaldehyde metabolism. These strains were cultured in MRS broth at 37°C for 3 days and harvested by centrifugation at 8,000 × g for 15 min at 4°C. For comparative analysis, *Lactobacillus brevis* K2 and *Lactobacillus fermentum* K4 were isolated from a commercially available hangover relief product in Korea. These strains were cultured and analyzed under the same conditions as the isolates from kimchi. All experimental procedures were conducted independently and adhered to ethical standards, complying with relevant legal and regulatory requirements. Thereafter, the harvested cells were washed three times with phosphate-buffered saline (PBS), followed by the resuspension of the strain in PBS to a final concentration of 5×10^8^ colony forming units (CFU)/100 µl and equal mixing for administration via gavage. The cell growth and density in liquid media were determined using a spectrophotometer (OD_600_).

### Genome analysis

Genomic DNA (gDNA) was extracted from actively growing liquid cultures of the strain during the logarithmic phase. Cell pellets were obtained by centrifugation and extracted using the DNeasy PowerSoil Pro Kit (Qiagen) following the manufacturer’s protocol. The quantity and quality of the gDNA were measured using a Qubit 4 fluorometer and a DS-11 spectrophotometer (DeNovix, USA) in Bio-Health Materials Core-Facility, Jeju National University.

Long-read sequencing libraries were prepared using the Native Barcoding Kit 24 V14 (SQK-NBD114.24), and sequencing was performed on the MinION sequencing platform (Oxford Nanopore Technology) with an R.10.4.1 flow cell (FLO-MIN114). Base-calling was conducted using MinKNOW (v23.04.6), and genome assembly was performed using the wf-bacterial-genomes pipeline embedded in EPI2ME. Annotation of the assembled genome was performed with the Prokka annotation pipeline (v1.14.6) ((Seemann, 2014). Homologous genes for metabolic pathways were BLAST-searched against non-redundant protein sequences in the KEGG (Kanehisa et al., 2016) and UniRef90 (Suzek et al., 2007) databases to identify genes involved in metabolic traits. Functional assignment of the predicted genes was improved by using a set of public databases [InterPro (Paysan-Lafosse et al., 2022), Gene Ontology (Ashburner et al., 2000; Consortium et al., 2023), Pfam (Mistry et al., 2021), the Conserved Domains Database (Wang et al., 2023), TIGRFAM (Selengut et al., 2007), and EggNOG (Hernández-Plaza et al., 2023)]. Prediction of signal peptides and transmembrane helices was performed by using the web-based services SignalP (v5.0) (Almagro Armenteros et al., 2019) and TMHMM (v2.0) (Krogh et al., 2001) with default settings. Finally, the presence of antibiotic-resistance genes was assessed using ResFinder (v4.3.3) (Ferrer Florensa et al., 2022).

Phylogenomic analysis was performed using the Anvi’o phylogenomics workflow (Eren et al., 2015) and the "Bacteria_71" single-copy marker gene set included in Anvi’o. A total of 49 genes, present in the genomes of the *Lactobacillaceae* family obtained from the NCBI, were selected for this analysis. The tree was constructed with IQ-TREE (v2.0.3) (-m MFP -B 1000) (Nguyen et al., 2014).

Average nucleotide identity (ANIb) was calculated by pyani (v0.2.13.1) (Pritchard et al., 2016) with BLASTn alignment. The resulting percentage identity and alignment coverage between genome pairs were presented in a table. Average amino acid identity (AAI) between genomes was determined using EzAAI (v1.2.1) (Kim et al., 2021).

### In vitro experiments

The alcohol tolerance of each lactic acid bacteria (LAB) strain was assessed using a modified method involving incubation in MRS broth containing 10% ethanol (v/v) (Kang et al., 2024; Yun et al., 2024). LAB cultures were initially centrifuged at 6,000 rpm for 10 min to remove the supernatant, followed by three washes with phosphate-buffered saline (PBS, pH 7.4). The resulting pellet was resuspended in MRS broth, and 99.9% EtOH (Merk Millipore) was added to reach a final ethanol concentration of 10% (v/v). The bacterial suspension was adjusted to a concentration of 10^9^ CFU/ml and inoculated into the ethanol-containing broth at 10% (v/v). Incubation occurred at 37°C for 4 h. Alcohol tolerance was quantified by measuring the optical density at 600 nm (OD_600_) (A1) and comparing the results to a control culture (A0). Alcohol tolerance was calculated using the following formula:

*Alcohol Tolerance* (%) = (*A1/A0*) × *100*

To evaluate the ethanol decomposition ability of LAB strains, ethanol was added to the bacterial suspensions at a final concentration of 2% (v/v). Similarly, the acetaldehyde decomposition capacity was assessed by incubating the LAB strains in MRS broth supplemented with acetaldehyde (CH_3_CHO) at a concentration of 8 mg per 50 ml. Each bacterial suspension (adjusted to 10^9^ CFU/ml) was inoculated into the ethanol or acetaldehyde-containing broth at a ratio of 1:15. The bacterial cultures were incubated at 37°C for 4 h. Ethanol concentrations in the culture supernatants were measured using the Ethanol Assay Kit (Megazyme, Ireland), while acetaldehyde concentrations were determined using the Acetaldehyde Assay Kit (Megazyme, Ireland), both according to the manufacturer’s instructions. The decomposition rates were calculated by comparing the ethanol or acetaldehyde concentrations in the culture supernatant with those in a blank control, where distilled water was used in place of the bacterial suspension.

All in vitro experiments were performed in triplicate, and data were expressed as mean ± standard deviation (SD). Statistical comparisons between groups were conducted using one-way ANOVA followed by Tukey's post hoc test for multiple comparisons. Statistical significance was defined as *p* < 0.05.

### In vivo experiments

To assess the impact of *L. suionicum* VITA-PB2 on ethanol and acetaldehyde metabolism, in vivo experiments were conducted using a Sprague-Dawley rat model. The study was conducted following the Laboratory Animal Act and was approved by the Institutional Animal Care and Use Committee (IACUC) of Jeju National University under protocol number approval ID # 2022-0057.

Male Sprague-Dawley rats, aged eight weeks and weighing between 250–300 g, were acquired from SAMTAKO BIO KOREA (Korea). Upon arrival, the rats were housed in a controlled environment with a temperature of 22°C ± 1°C, humidity of 50% ± 10%, and a 12-h light/dark cycle. They were acclimated for one week, during which they had unrestricted access to standard chow and water. On the final day of treatment, the rats were fasted for 18 h with only water provided. The rats were randomly divided into three groups (n = 7–8 per group): a control group that received 200 µl (2 ml/kg) of saline; a negative group that was administered with 25% ethanol (10 ml/kg) after 30 min of the experiment began, and a VITA-PB2 group that was administered 10^9^ cells of strain VITA-PB2 by orally, followed by 25% ethanol (10 ml/kg) 30 min after the experiment began. The bacterial strain was cultured in MRS broth, harvested, and resuspended in phosphate-buffered saline (PBS) to achieve the desired concentrations. Blood samples were collected at 1, 3, and 5 h post-ethanol administration. The rats were anesthetized using isoflurane (Virbac, France) to minimize distress, and blood was collected from the jugular vein. The collected blood was centrifuged at 3,000 rpm for 15 min at 4°C, and the plasma was stored at -70°C for further analysis.

At 6 h post-ethanol administration, the rats were sacrificed, and liver tissues were excised. The liver samples were snap-frozen in liquid nitrogen and stored at -70°C for subsequent enzymatic assays. Other portions of the liver were fixed in 10% neutral-buffered formalin for histological analysis. Blood ethanol and acetaldehyde concentrations were measured using enzymatic assay kits (see above). To assess the activity of ADH and quantify ALDH in liver tissues, the samples were homogenized in cold PBS and then centrifuged at 10,000 rpm for 20 min at 4°C. The supernatant was collected, and protein concentrations were determined using the Bradford assay. The activities of ADH and the quantification of ALDH were measured using specific assay kits (ADH-Ab102533 and ALDH-RK14173).

All data were expressed as mean ± standard deviation (SD). Statistical analyses were performed using one-way ANOVA followed by Tukey's post hoc test for multiple comparisons, with a significance level set at *p* < 0.05. All analyses were conducted using GraphPad Prism version 8.02.263 (USA).

## Result and Discussion

### Phylogenetic and genomic characterization of LAB strains

*Pediococcus pentosaceus* VITA-PK1 and *Leuconostoc suionicum* VITA-PB2 are characterized as Gram-positive, facultatively anaerobic, non-motile, and non-sporulating LAB strains isolated from traditional Korean Kimchi. To investigate the phylogenetic properties and genomic characteristics of these LAB strains, we performed genome sequencing analysis. The draft genome of VITA-PK1 is composed of a single contig, with a total genome size of 1,719,447 bp with a coverage depth of 148×. The VITA-PB2 genome consists of four contigs, with a total genome size of 2,193,477 bp with a coverage depth of 100×.

Phylogenomic analysis identified strains VITA-PK1 and VITA-PB2 as *Pediococcus pentosaceus* and *Leuconostoc suionicum*, respectively (Fig. S1), with confirmation from ANI and AAI analyses (Table S1–4). Strain VITA-PB2, initially classified as *Leuconostoc mesenteroides*, was reclassified as *Leuconostoc suionicum* following its designation as a distinct species in 2017 (Jeon et al., 2017). The G+C content of *P. pentosaceus* VITA-PK1 (37.29%) and *L. suionicum* VITA-PB2 (37.11%) were consistent with the known ranges for their respective genera: *Pediococcus* (37–42%) and *Leuconostoc* (37–38%). The strains contained 1,851 and 2,209 coding sequences (CDSs), respectively.

The ANIb values for *P. pentosaceus* VITA-PK1 ranged from 72.5% to 99.7% compared to other members of the genus *Pediococcus*, with the highest value (99.7% ANIb and 90.7% coverage) observed with *P. pentosaceus* CECT 8330 (Table S1). The AAI values ranged from 65.3% to 99.6%, with the highest value also observed with *P. pentosaceus* CECT 8330 (Table S2). For *L. suionicum* VITA-PB2, the ANIb values ranged from 74.0% to 98.9%, with the highest value (98.9% ANIb and 87.3% coverage) observed with *L. suionicum* DSM 20241 (Table S3). The AAI values ranged from 70.5% to 99.1%, with the highest value observed with *L. suionicum* UCMA20148 (Table S4).

*P. pentosaceus* CECT 8330, closely related to strain VITA-PK1, is a probiotic strain known for its role in improving gut health, particularly through modulating the gut microbiota and enhancing short-chain fatty acid metabolism (Chen et al., 2021; Dong et al., 2022; Santas et al., 2015). Recent studies have also demonstrated its potential to alleviate ethanol-induced liver injury by stabilizing gut integrity and reducing inflammation markers (Jiang et al., 2020). Similarly, *L. suionicum* DSM 20241, closely related to strain VITA-PB2, is recognized for its role in the fermentation of foods like kimchi and its ability to support gut health (Chun et al., 2017; Jeon et al., 2017). Genomic studies indicate its capability to synthesize essential metabolites, contributing to its probiotic potential. Furthermore, related strains such as *L. mesenteroides* WiKim0172 have shown alcohol tolerance and the ability to enhance alcohol metabolism by increasing ADH and ALDH activity (Yun et al., 2024), suggesting the broader utility of *Leuconostoc* species in mitigating alcohol-induced damage.

**Fig. 1.**
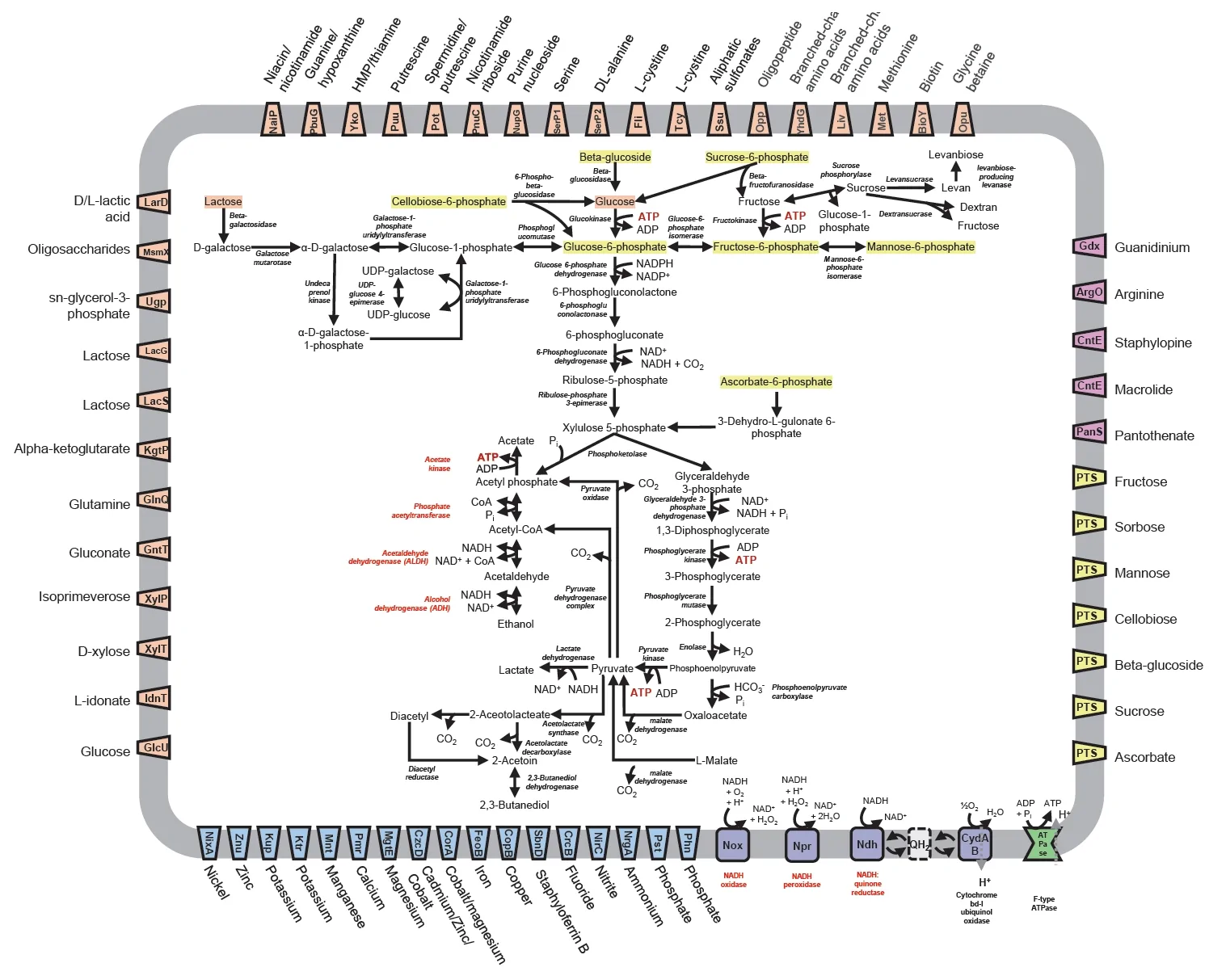
Genomic metabolic pathways identified in *Leuconostoc suionicum* VITA-PB2. Enzymes involved in the aerobic metabolic pathways for ethanol and acetaldehyde consumption are highlighted in red. The pathway between ethanol and acetate is referenced by Tagaino et al. (2019), and Fukui et al. (1988), and the KEGG pathway. Graphical representation of metabolic pathways for carbon utilization, with focus on pentose phosphate, fructose, and mannose pathways. Gene presence for phosphotransferase system (PTS), enabling carbon source utilization and fermentation processes. UDP: uridine diphosphate.

Strain VITA-PB2 employs a heterolactic fermentative pathway, converting sugars into lactic acid, ethanol, and CO₂. It utilizes metabolic pathways such as pentose phosphate, fructose and mannose, and pyruvate metabolism, but lacks a complete tricarboxylic acid (TCA) cycle (Fig. 1; Table S5 and Dataset S2). Genes for the phosphotransferase system (PTS) enable the utilization of diverse carbon sources like fructose, mannose, and sucrose, which are processed into lactate, ethanol, or acetate with CO₂ as a byproduct (Fig. 1; Dataset S1). VITA-PB2 also synthesizes polysaccharides like levanbiose and dextran (Fig. 1; Table S5 and Dataset S1), offering probiotic benefits such as pathogen inhibition and stress protection (Pramudito et al., 2024; Yokota et al., 1993). Like other *Leuconostoc* spp., it contains a gene set associated with bacteriocin production (Table S5 and Dataset S1).

Upon analyzing the genes involved in alcohol and acetaldehyde degradation, the VITA-PK1 genome was found to contain three ADH genes, with EC number 1.1.1.- (VITAPK1_00365, VITAPK1_00541, and VITAPK1_00710), but lacks genes encoding ALDH, as indicated in Table S1 and Dataset S1. In contrast, the VITA-PB2 genome harbors four ADH genes, along with a bifunctional acetaldehyde-CoA/alcohol dehydrogenase with EC number EC 1.2.1.10 and 1.1.1.1, as well as an ALDH gene (EC 1.2.1.3), as indicated in Table S1 and Dataset S2. This genetic composition suggests that strain VITA-PB2 has potential to metabolize ethanol and acetaldehyde effectively.

Strain VITA-PB2 lacks key genes for a complete electron transport chain (ETC) (Fig. 1; Table S5 and Dataset S1), including Complex III (EC 7.1.1.8) and catalase (EC 1.11.1.6), making it sensitive to oxygen exposure. To mitigate oxidative stress, VITA-PB2 relies on a high-affinity cytochrome *bd* complex (CydA and CydB) and a thiol-exporting ABC transporter (CydDC) instead of the more efficient cytochrome c oxidase (Fig. 1; Table S5 and Dataset S1). This cytochrome *bd* complex reduces oxygen to water with lower proton-pumping efficiency, enabling survival in low-oxygen or microaerophilic conditions, such as fermentation environments (Borisov et al., 2011). Additionally, VITA-PB2 harbors genes for NADH:quinone reductase, NAD(P)/FAD-dependent oxidoreductases, NADH-dependent oxidoreductase, NADH peroxidase, NADH oxidase and NADH-dependent flavin oxidoreductase (Fig. 1; Table S5 and Dataset S1), which facilitate NAD^+^ regeneration and aid in maintaining the NADH/NAD^+^ balance (Higuchi et al., 1999; Koike et al., 1985). These mechanisms, combined with the cytochrome *bd* complex, help mitigate reactive oxygen species production (Sena et al., 2024). However, the absence of Complex I and a complete TCA cycle may limit the ability of strain VIITA-PB2 to thrive in highly oxygenated environments (Shin & Han, 2015).

### Alcohol metabolism in in vitro test

**Fig. 2.**
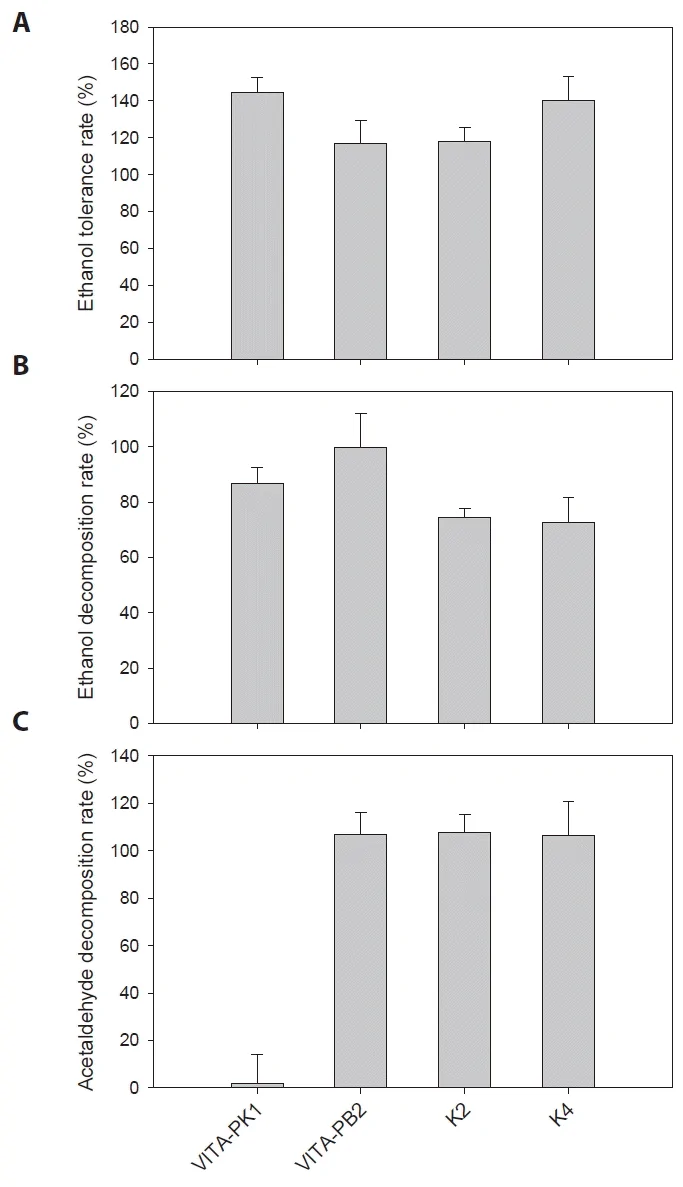
Ethanol and acetaldehyde decomposition abilities of LAB strains in vitro. (A) Alcohol tolerance levels of four LAB strains, including strain VITA-PB2, after incubation in ethanol-containing media. (B) Ethanol decomposition rates of the tested strains, with strain VITA-PB2 exhibiting the highest rate of ethanol breakdown. (C) Acetaldehyde decomposition rates show the strong capability of strain VITA-PB2 to detoxify acetaldehyde compared to control strains. Values are presented as the mean ± standard deviation from triplicate experiments.

The in vitro experiments were conducted to evaluate the alcohol tolerance and the ethanol and acetaldehyde decomposition abilities of 2 isolated strains (*P. pentosaceus* VITA-PK1 and *L. suionicum* VITA-PB2) and 2 control strains (*Lactobacillus brevis* K2 and *Lactobacillus fermentum* K4). The results from the alcohol tolerance test indicated that all four LAB strains demonstrated significant resistance to ethanol, with ethanol tolerance values exceeding 100% within 4 h (Fig. 2A). This suggests that the LAB strains we tested in this study can survive and potentially function in high-alcohol environments, which is crucial for their application in fermentation processes or in managing alcohol metabolism in food products.

The ethanol decomposition test further demonstrated the metabolic capabilities of these LAB strains. As shown in Fig. 2B, strain VITA-PB2 was the most efficient, achieving an ethanol decomposition rate of ~100%. This capacity of strain VITA-PB2 to rapidly convert ethanol into acetaldehyde and subsequently into non-toxic byproducts such as acetate suggests its potential as a candidate for the development of functional foods aimed at lowering blood alcohol levels and mitigating the toxic effects of alcohol. While it is known that different bacterial strains produce varying concentrations of organic acids (Martino et al., 2022; Tagaino et al., 2019), the specific concentrations of acetate and other organic acids were not quantified in this study, leaving the precise end-products of acetaldehyde decomposition uncertain. Other tested strains also exhibited ethanol decomposition rates exceeding 70%. ADH is a group of enzymes present in many organisms that catalyze the interconversion of alcohols and aldehydes or ketones, accompanied by the reduction of nicotinamide adenine dinucleotide (NAD^+^) to NADH under mostly aerobic conditions (Wu et al., 2021). The presence of ADH genes in strains VITA-PB2 and VITA-PK1 likely contributes to their superior ethanol decomposition abilities, as ADH facilitates ethanol oxidation to acetaldehyde.

The acetaldehyde decomposition capabilities of the LAB strains were evaluated, and the results are presented in Fig. 2C. Strain VITA-PB2 demonstrated a remarkable ability to decompose acetaldehyde, higher than 100% decomposition within the 4-h incubation period. *L. brevis* K2 and *L. fermentum* K4 also exhibited high acetaldehyde decomposition rates, while strain VITA-PK1 showed almost no activity in acetaldehyde breakdown because of the absence of ALDH gene in the genome. The strong acetaldehyde decomposition ability of strain VITA-PB2 can be attributed to the presence of ALDH genes identified in the genome. The ability to efficiently convert acetaldehyde is a key factor in reducing the toxic burden of alcohol consumption, thereby mitigating hangover symptoms and other adverse effects associated with alcohol metabolism (Fukui et al., 1988; Konkit et al., 2015; Tagaino et al., 2019).

The ALDH enzyme plays a crucial role in detoxifying acetaldehyde during alcohol metabolism by catalyzing its oxidation to acetate through a two-step reaction that also requires NAD^+^ as a cofactor, with the activity of the enzyme being tightly regulated by the availability of cofactors and its structural integrity; studies have shown that ALDH forms high-order assemblies like spirosomes, which facilitate efficient substrate channeling and enzymatic turnover, especially under conditions of high acetaldehyde concentration, such as excessive alcohol consumption, thus preventing the accumulation of toxic acetaldehyde and mitigating its harmful effects, including liver damage and hangover symptoms (Fukui et al., 1988; Konkit et al., 2015; Tagaino et al., 2019).

The results of this study align with previous findings that have highlighted the importance of strain-specific capabilities in LAB for alcohol metabolism. For example, *Limosilactobacillus fermentum* has been shown to significantly reduce blood alcohol levels and enhance liver function by boosting ADH and ALDH activities (Kim et al., 2003). Similarly, the robust performance of *Leuconostoc suionicum* VITA-PB2 in both ethanol and acetaldehyde decomposition underscores the potential of certain LAB strains to serve as effective agents in functional foods or supplements designed to mitigate the toxic effects of alcohol consumption (Yun et al., 2024). The strain-specific nature of these metabolic abilities emphasizes the need for careful selection of LAB strains based on their genetic and enzymatic profiles.

Collectively, the in vitro tests demonstrate that strain VITA-PB2 is particularly effective in alcohol metabolism, exhibiting strong resistance to ethanol and remarkable capabilities in decomposing both ethanol and acetaldehyde. Therefore, while these in vitro results are promising, additional experiments were necessary to verify the alcohol degradation ability of strain VITA-PB2 in vivo, as the complexities of the biological environment can influence how effectively such mechanisms function in a living organism.

### Alcohol metabolism in in vivo test

**Fig. 3.**
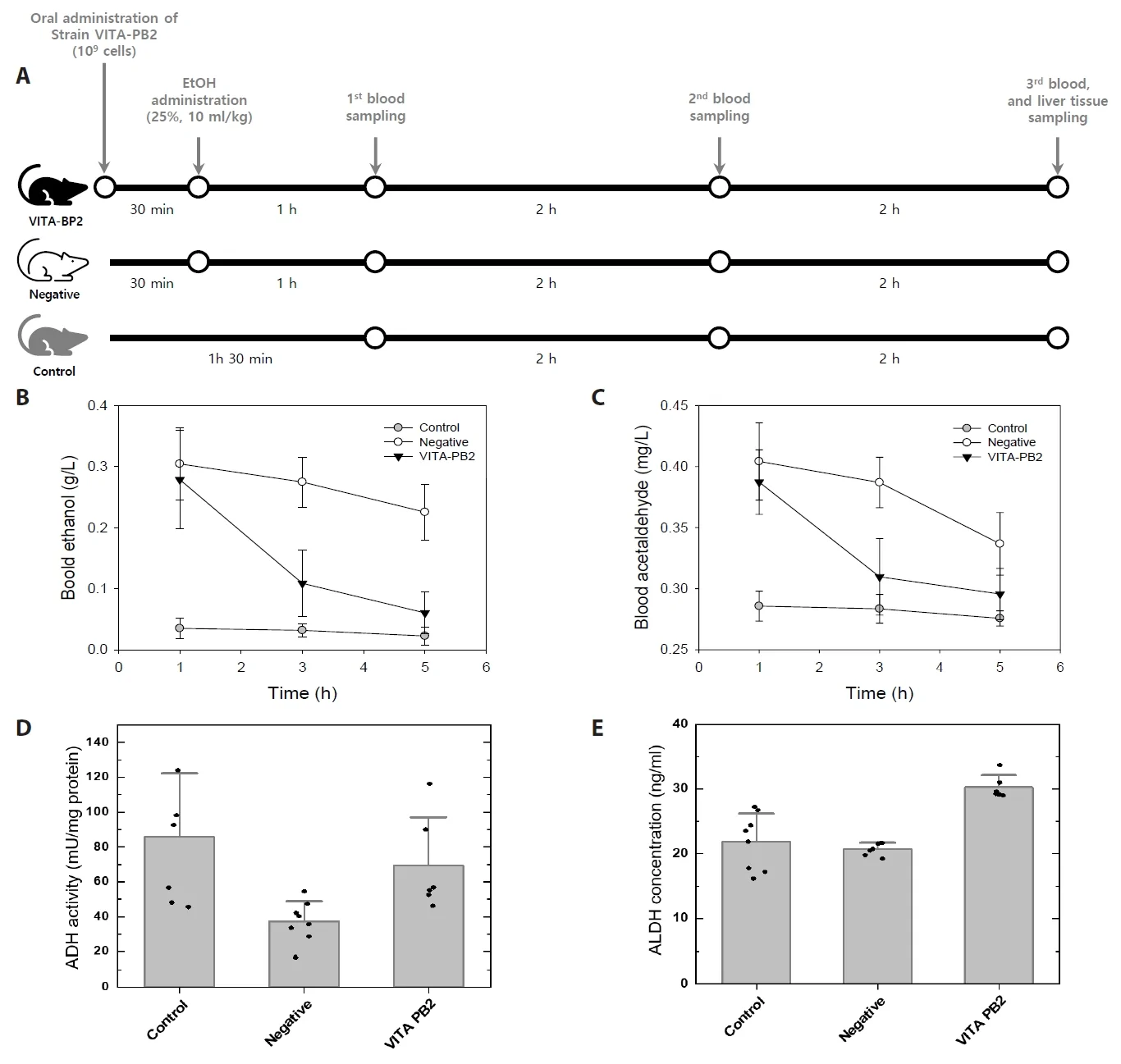
In vivo effects of *Leuconostoc suionicum* VITA-PB2 on ethanol and acetaldehyde metabolism in rats. (A) Experimental design for in vivo effects of *Leuconostoc suionicum* VITA-PB2 on alcohol metabolism in rats. The schematic illustrates the experimental timeline, including the administration of VITA-PB2 followed by ethanol treatment. Blood samples were collected at 1, 3, and 5 h post-ethanol administration, and liver tissue harvested at the 5 h mark. The experimental groups were defined as follows: control, untreated with VITA-PB2 and ethanol; negative, treated with ethanol but not VITA-PB2; VITA-PB2, treated with VITA-PB2 and ethanol. (B) Blood ethanol and (C) Blood acetaldehyde concentrations; (D) Hepatic alcohol dehydrogenase (ADH) activity and (E) aldehyde dehydrogenase (ALDH) expression were measured from liver samples collected after 5 hhour. Values are presented as the mean ± standard deviation from triplicate experiments. Significant differences between groups were determined by a t-test (^*^p < 0.05). A higher *p*-value of 0.05 was considered not significant.

The efficacy of *L. suionicum* VITA-PB2 in modulating alcohol metabolism was evaluated through in vivo experiments using a Sprague-Dawley rat model (Fig. 3A). The primary focus was on assessing the impact of this LAB strain on blood ethanol and acetaldehyde concentrations, as well as the activities of ADH and ALDH in liver tissues, following ethanol administration.

Following ethanol administration, the ethanol-treated groups (VITA-PB2 and negative groups) exhibited a typical ethanol metabolism curve, with peak blood ethanol levels observed at approximately 1-h post-administration (Fig. 3B). In contrast, the groups administered with strain VITA-PB2 showed a significant reduction in blood ethanol levels compared to the control group. At the 3 h, the VITA-PB2 group showed a 68% reduction in blood ethanol concentration relative to the negative group (*p* < 0.05). This reduction further increased to 81% by the 5 h (*p* < 0.05), suggesting a potential role of VITA-PB2 in reducing blood alcohol levels.

In terms of acetaldehyde, which is a toxic by-product of ethanol metabolism (see above), the control group maintained elevated acetaldehyde levels throughout the observation period, reflecting the normal metabolic process of ethanol to acetaldehyde and its subsequent accumulation. However, in the VITA-PB2 groups, blood acetaldehyde levels were significantly lower than in the negative group: the VITA-PB2 group exhibited a 75% reduction in acetaldehyde levels at 3 h and a 67% reduction at 5 h post-ethanol administration (*p* < 0.05) (Fig. 3C). These results suggest that strain VITA-PB2 not only accelerates ethanol metabolism but also facilitates the rapid detoxification of acetaldehyde, converting it into acetate more efficiently.

To further understand the metabolic impacts of strain VITA-PB2 administration, the activity of ADH and expression of ALDH enzymes in liver tissues were measured. The results revealed a significantly higher activity of ADH and expression of ALDH in the VITA-PB2 groups than in the negative group (Fig. 3D & 3E). ADH activity in the VITA-PB2 group showed a non-significant reduction compared to the control group (*p* > 0.05), but it was 1.75-fold higher than that in the negative group (*p* < 0.05) (Fig. 3D). In contrast, expression of ALDH in the VITA-PB2 group was approximately 1.5-fold higher than in the control and negative groups (*p* < 0.05) (Fig. 3E). The ADH activity in liver tissues could decrease following excessive ethanol exposure (He et al., 2002; Tran et al., 2015). The difference in ADH activity in liver tissues between the VITA-PB2 group and the negative group, both of which were orally administered alcohol, along with the reduction in blood ethanol levels observed in Fig. 3B, suggests that the liver tissues of the VITA-PB2 group may have been less exposed to alcohol due to the intrinsic ADH activity of the administered strain and/or the gut microbiota. This study aligns with previous findings by Yun et al. (2024) and Lu et al. (2020), demonstrating that LAB strains significantly contribute to ethanol and acetaldehyde metabolism. The increase in ALDH expression observed in the VITA-PB2 group indicates that administration of LAB not only prevents the acetaldehyde accumulation within microbial cells but also enhances hepatic ALDH expression in the host. This enhancement facilitates more efficient detoxification and improved liver function (Gan et al., 2021; Kim et al., 2003).

LAB strains, such as *Leuconostoc*, utilize ADH in ethanol-tolerant environments to oxidize ethanol into acetaldehyde during reverse fermentation (Jung et al., 2021; Konkit et al., 2015). Meanwhile, mammalian ADH and ALDH enzymes are highly compartmentalized and tightly regulated in liver tissues (Deltour et al., 1999), LAB enzymes operate within broader fermentation networks, responding dynamically to environmental ethanol and acetaldehyde concentrations (Fukui et al., 1988; Lu et al., 2020; Salaspuro, 1997; Tagaino et al., 2019). In this study, strain VITA-PB2 demonstrated a dual role in ethanol metabolism: microbial ADH facilitated ethanol breakdown, and elevated ALDH activity efficiently reduced acetaldehyde levels in both microbial systems and host liver tissues (Fig. 3). This synergistic action accelerated ethanol clearance and mitigated acetaldehyde toxicity, aligning with previous findings that LAB administration enhances ALDH activity and improves liver function (Kim et al., 2003; Lu et al., 2020; Yun et al., 2024). The increased activity of ADH and expression of ALDH in liver tissues observed in the VITA-PB2 group provide mechanistic evidence for the reduced blood ethanol and acetaldehyde levels (Fig. 3). These results underscore the potential of strain VITA-PB2 as a functional probiotic capable of protecting against alcohol-induced liver damage and related chronic conditions, such as fatty liver disease and hepatic cirrhosis.

## Conclusion

This study demonstrates the significant potential of *Leuconostoc suionicum* VITA-PB2 in enhancing alcohol metabolism, positioning it as a promising candidate for functional foods or supplements aimed at mitigating the toxic effects of alcohol consumption. Both in vitro and in vivo experiments confirmed the efficacy of strain in reducing blood ethanol and acetaldehyde levels, highlighting its ability to accelerate alcohol clearance and detoxify harmful by-products. The increased expression of ALDH observed in the VITA-PB2-treated groups, along with its intrinsic ADH activity, suggests that the strain not only assists in ethanol breakdown but also enhances the detoxification of host pathways. This dual mechanism makes strain VITA-PB2 an effective probiotic for improving alcohol metabolism and reducing symptoms such as hangovers. Given its superior performance, particularly in decomposing acetaldehyde, strain VITA-PB2 offers a novel approach to managing the adverse effects of alcohol consumption, such as hangovers and alcohol-induced liver damage. These findings suggest that regular administration of strain VITA-PB2 could provide long-term protective effects, potentially preventing chronic alcohol-related conditions such as fatty liver disease and hepatic cirrhosis. Overall, the strain-specific advantages of the LAB strain emphasize the need for targeted probiotic applications in alcohol metabolism and lay the groundwork for future studies focusing on its broader health benefits.

## Supplementary Information

Table S1. The pairwise comparison of average nucleotide identity (ANI) values for the genomes of Pediococcus pentosaceus VITA-PK1 and reltated strains.

Table S2. The pairwise comparison of Average Amino Acid Identity (AAI) values for the genomes of Pediococcus pentosaceus VITA-PK1 and related strains.

Table S3. The pairwise comparison of average nucleotide identity (ANI) values for the genomes of Leuconostoc suionicum VITA-PB2 and reltated strains.

Table S4. The pairwise comparison of Average Amino Acid Identity (AAI) values for the genomes of Leuconostoc suionicum VITA-PB2 and related strains.

Table S5. Genes of interest identified in the VITA-PB2 genome. The locus IDs matched those in Dataset S1.

Fig. S1. Phylogenomic tree and average nucleotide and amino acid identity (ANI and AAI) matrices for the genomes of *Pediococcus pentosaceus* VITA-PK1 and *Leuconostoc suionicum* VITA-PB2 strains, along with related species within the family *Lactobacillaceae*. Phylogenomic tree constructed based on 49 genes from the "Bacteria_71" single-copy marker gene set using Anvi’o’s phylogenomic workflow. The maximum-likelihood phylogeny was inferred using IQ-TREE (options: -B 1000 -m LG+F+I+R6). Black circles at the nodes indicate bootstrap support of 70% or higher.

Dataset S1. List of predicted genes in Leuconostoc suionicum VITA-PB2.

Dataset S2. List of predicted genes in Pediococcus pentosaceus VITA-PK1.
