# Supplementary material for: Comprehensive genomic and functional analysis of Leuconostoc lactic acid bacteria in alcohol and acetaldehyde metabolism
Source: J Microbiol. 2025 Feb 27;63(2):e2410026. doi: 10.71150/jm.2410026 (PMC13577034; doi:10.71150/jm.2410026)
Supplement: Supplementary file 1 [file jm-2410026-Supplementary-Fig-S1.pdf]

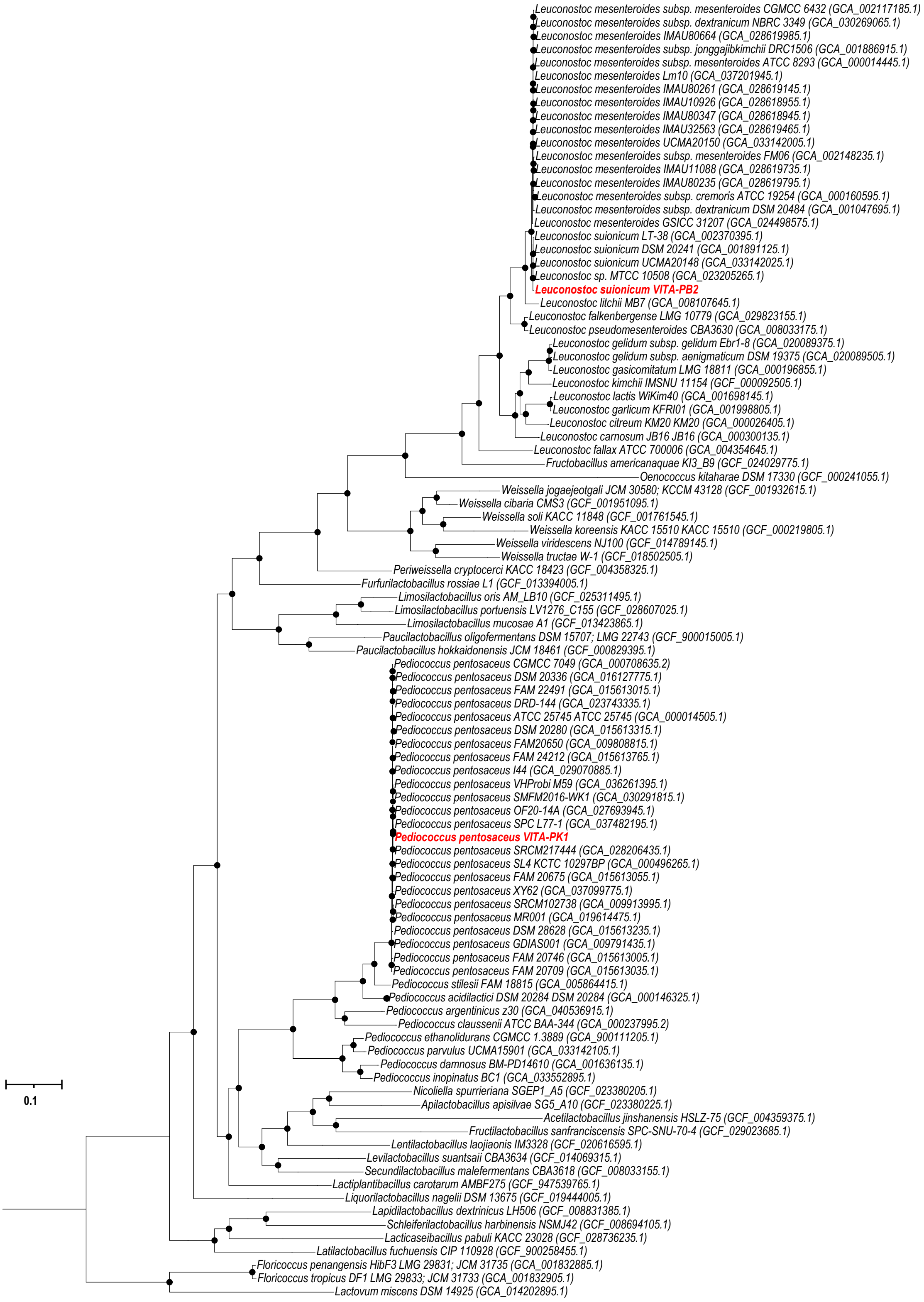

Fig. S1. Phylogenomic tree and average nucleotide and amino acid identity (ANI and AAI) matrices for the genomes of *Pediococcus pentosaceus* VITA-PK1 and *Leuconostoc suionicum* VITA-PB2 strains, along with related species within the family *Lactobacillaceae*. Phylogenomic tree constructed based on 49 genes from the "Bacteria_71" single-copy marker gene set using Anvi'o's phylogenomic workflow. The maximum-likelihood phylogeny was inferred using IQ-TREE (options: -B 1000 -m LG+F+I+R6). Black circles at the nodes indicate bootstrap support of 70% or higher.
