# Supplementary material for: Comprehensive genomic and functional analysis of Leuconostoc lactic acid bacteria in alcohol and acetaldehyde metabolism
Source: J Microbiol. 2025 Feb 27;63(2):e2410026. doi: 10.71150/jm.2410026 (PMC13577034; doi:10.71150/jm.2410026)
Supplement: Supplementary file 2 [file jm-2410026-Supplementary-Table-S1.pdf]

Table S1. The pairwise comparison of average nucleotide identity (ANI) values for the genomes of *Pediococcus pentosaceus* VITA-PK1 and reltated strains.

Genome	1	2	3	4	5	6	7	8	9	10	11	12	13	14	15	16
1. *P. acidilactici* DSM 20284	**100**															
2. *P. acidilactici* DSM 20238	**99.0**	**100**														
3. *P. acidilactici* DSM 19927	**97.0**	**97.1**	**100**													
4. *P. acidilactici* KCTC 14696BP	**97.2**	**97.2**	**98.8**	**100**												
5. *P. pentosaceus* DSM 20280	75.6	75.1	75.4	75.8	**100**											
6. *P. pentosaceus* DSM 46292	75.4	75.2	75.1	75.5	**98.6**	**100**										
7. *P. pentosaceus* DSM 28628	75.4	75.2	75.2	75.4	**98.6**	**98.9**	**100**									
8. *P. pentosaceus* DSM 20283	75.7	75.4	75.4	75.7	**98.6**	**98.8**	**99.1**	**100**								
9. *P. pentosaceus* CECT 8330; DSM 32537	75.5	75.2	75.3	75.5	**98.6**	**98.9**	**99.1**	**99.9**	**100**							
**10. *P. pentosaceus* VITA-PK1**	75.6	75.4	75.3	75.5	**98.6**	**98.8**	**99.0**	**99.7**	**99.7**	**100**						
11. *P. pentosaceus* SL4 KCTC 10297BP	75.5	75.3	75.3	75.5	**98.5**	**98.7**	**99.0**	**99.7**	**99.8**	**99.4**	**100**					
12. *P. pentosaceus* DSM 20336	75.7	75.5	75.5	76.0	**98.6**	**98.8**	**99.0**	**99.0**	**99.0**	**98.9**	**98.9**	**100**				
13. *P. claussenii* DSM 14800	72.9	72.9	72.7	73.2	74.1	73.7	73.5	74.0	73.7	73.7	73.7	73.8	**100**			
14. *P. argentinicus* DSM 23026	73.7	73.7	73.5	73.8	74.9	74.7	74.3	74.8	74.6	74.5	74.4	74.6	74.2	**100**		
15. *P. ethanolidurans* CIRM-BIA2669	72.8	72.6	72.4	73.0	74.0	73.3	72.7	73.4	73.0	73.3	73.0	73.3	73.1	73.4	**100**	
16. *P. inopinatus* DSM 20285	72.9	72.7	72.3	73.5	73.9	73.4	72.7	73.4	73.0	73.1	73.1	73.3	73.2	73.1	78.2	**100**

*Note: The table shows the average nucleotide identity (ANI) percentages among the genomes of *Pediococcus pentosaceus* VITA-PK1 and related strains. Each rows and columns represent individual genomes. Values exceeding 95% are shown in bold. *Pediococcus pentosaceus* VITA-PK1 highlighted in red.
