# Supplementary material for: Comprehensive genomic and functional analysis of Leuconostoc lactic acid bacteria in alcohol and acetaldehyde metabolism
Source: J Microbiol. 2025 Feb 27;63(2):e2410026. doi: 10.71150/jm.2410026 (PMC13577034; doi:10.71150/jm.2410026)
Supplement: Supplementary file 3 [file jm-2410026-Supplementary-Table-S2.pdf]

Table S2. The pairwise comparison of Average Amino Acid Identity (AAI) values for the genomes of *Pediococcus pentosaceus* VITA-PK1 and related strains.

**Genome**	**1**	**2**	**3**	**4**	**5**	**6**	**7**	**8**	**9**	**10**	**11**	**12**	**13**	**14**	**15**	**16**
1. *P. ethanolidurans* CIRM-BIA2669	**100**															
2. *P. inopinatus* DSM 20285	82.7	**100**														
3. *P. claussenii* DSM 14800	66.0	66.1	**100**													
4. *P. argentinicus* DSM 23026	66.9	66.5	70.9	**100**												
**5. *P. pentosaceus* VITA-PK1**	65.7	65.7	70.2	70.5	**100**											
6. *P. pentosaceus* SL4 KCTC 10297BP	65.6	65.6	70.3	70.6	**99.2**	**100**										
7. *P. pentosaceus* DSM 20283	65.6	65.9	70.3	70.7	**99.6**	**99.5**	**100**									
8. *P. pentosaceus* CECT 8330; DSM 32537	65.7	65.8	70.3	70.9	**99.6**	**99.6**	**99.9**	**100**								
9. *P. pentosaceus* DSM 20336	65.7	65.9	70.4	70.7	**99.0**	**99.0**	**99.1**	**99.2**	**100**							
10. *P. pentosaceus* DSM 28628	65.8	65.7	70.3	70.7	**99.0**	**98.9**	**99.0**	**99.1**	**99.1**	**100**						
11. *P. pentosaceus* DSM 46292	65.9	66.1	70.3	70.7	**98.7**	**98.5**	**98.8**	**98.9**	**98.8**	**98.7**	**100**					
12. *P. pentosaceus* DSM 20280	66.1	66.0	70.6	70.9	**98.4**	**98.4**	**98.7**	**98.7**	**98.6**	**98.6**	**98.3**	**100**				
13. *P. acidilactici* DSM 20284	65.4	65.5	69.8	70.5	78.4	78.4	78.4	78.4	78.5	78.3	78.1	78.1	**100**			
14. *P. acidilactici* DSM 20238	65.6	65.6	70.2	70.4	78.4	78.4	78.3	78.3	78.4	78.2	78.1	77.8	**99.0**	**100**		
15. *P. acidilactici* KCTC 14696BP	65.6	65.9	70.0	70.4	78.3	78.1	78.3	78.3	78.5	78.1	78.1	78.1	**98.0**	**98.0**	**100**	
16. *P. acidilactici* DSM 19927	65.7	65.6	70.0	70.5	78.4	78.3	78.4	78.4	78.5	78.2	78.0	78.1	**98.0**	**98.0**	**98.9**	**100**

*Note: The table shows the average amino acid identity (AAI) percentages among the genomes of *Pediococcus pentosaceus* VITA-PK1 and closely related taxa. Each rows and columns represent individual genomes. Values exceeding 95% are shown in bold. *Pediococcus pentosaceus* VITA-PK1 highlighted in red.
