# Supplementary material for: Comprehensive genomic and functional analysis of Leuconostoc lactic acid bacteria in alcohol and acetaldehyde metabolism
Source: J Microbiol. 2025 Feb 27;63(2):e2410026. doi: 10.71150/jm.2410026 (PMC13577034; doi:10.71150/jm.2410026)
Supplement: Supplementary file 4 [file jm-2410026-Supplementary-Table-S3.pdf]

Table S3. The pairwise comparison of average nucleotide identity (ANI) values for the genomes of *Leuconostoc suionicum* VITA-PB2 and reltated strains.

**Genome**	**1**	**2**	**3**	**4**	**5**	**6**	**7**	**8**	**9**	**10**	**11**	**12**	**13**	**14**	**15**	**16**
**1. *L. suionicum* VITA-PB2**	**100**															
2. *L. suionicum* DSM 20241	**98.9**	**100**														
3. *L. suionicum* LT-38	**98.8**	**100**	**100**													
4. *L*. sp. MTCC 10508	**99.0**	**99.5**	**99.5**	**100**												
5. *L. suionicum* UCMA20148	**98.9**	**99.5**	**99.5**	**99.5**	**100**											
6. *L. mesenteroides* DSM 20346	94.0	93.9	94.0	93.9	93.9	**100**										
7. *L. cremoris* ATCC 19254	94.0	94.0	94.0	94.0	94.0	**100**	**100**									
8. *L. dextranicum* DSM 20484	94.2	94.2	94.2	94.2	94.1	**99.0**	**99.0**	**100**								
9. *L. mesenteroides* ATCC 8293	93.8	93.9	93.9	93.7	93.9	**98.7**	**98.7**	**98.9**	**100**							
10. *L. mesenteroides* ATCC 8293D-5	93.8	93.9	93.9	93.7	93.9	**98.7**	**98.7**	**98.9**	**100**	**100**						
11. *L. dextranicum* NBRC 3349	93.9	93.9	94.0	93.8	93.8	**98.6**	**98.6**	**98.9**	**99.1**	**99.1**	**100**					
12. *L. aenigmaticum* DSM 19374	74.6	74.8	74.7	74.5	74.6	74.5	74.7	74.5	74.7	74.7	74.7	**100**				
13. *L. carnosum* DSM 5576	75.1	75.3	74.9	74.9	75.2	75.0	75.0	75.1	75.3	75.5	75.2	76.8	**100**			
14. *L. fallax* ATCC 700006	74.2	73.6	73.7	73.8	73.8	73.7	73.8	73.8	73.7	73.8	73.7	73.5	74.2	**100**		
15. *L. falkenbergense* DSM 5624	76.5	76.9	76.8	76.0	76.1	76.7	76.7	76.9	76.7	76.8	77.1	75.0	75.3	74.0	**100**	
16. *L. pseudomesenteroides* DSM 20193	76.2	76.0	76.1	75.8	75.9	76.1	76.1	76.1	76.1	76.2	76.0	74.6	74.9	73.7	92.3	**100**

*Note: The table shows the Average Nucleotide Identity (ANI) percentages among the genomes of *Leuconostoc suionicum* VITA-PB2 and related strains. Each rows and columns represent individual genomes.Values exceeding 95% are shown in bold. *Leuconostoc suionicum* VITA-PB2 highlighted in red.
