# Supplementary material for: Comprehensive genomic and functional analysis of Leuconostoc lactic acid bacteria in alcohol and acetaldehyde metabolism
Source: J Microbiol. 2025 Feb 27;63(2):e2410026. doi: 10.71150/jm.2410026 (PMC13577034; doi:10.71150/jm.2410026)
Supplement: Supplementary file 5 [file jm-2410026-Supplementary-Table-S4.pdf]

Table S4. The pairwise comparison of Average Amino Acid Identity (AAI) values for the genomes of *Leuconostoc suionicum* VITA-PB2 and related strains.

Genome	1	2	3	4	5	6	7	8	9	10	11	12	13	14	15	16
1. *L.* sp. MTCC 10508	**100**															
2. *L. suionicum* UCMA20148	**99.6**	**100**														
**3. *L. suionicum* VITA-PB2**	**99.0**	**99.1**	**100**													
4. *L. suionicum* DSM 20241	**99.3**	**99.5**	**98.7**	**100**												
5. *L. suionicum* LT-38	**99.3**	**99.5**	**98.7**	**100**	**100**											
6. *L. mesenteroides* DSM 20346	**95.7**	**95.9**	**95.7**	**95.7**	**95.7**	**100**										
7. *L. mesenteroides* ATCC 19254	**95.7**	**95.9**	**95.7**	**95.7**	**95.7**	**100**	**100**									
8. *L. mesenteroides* DSM 20484	**95.8**	**95.9**	**95.9**	**95.9**	**95.9**	**98.6**	**98.6**	**100**								
9. *L. mesenteroides* NBRC 3349	**95.5**	**95.5**	**95.6**	**95.3**	**95.4**	**98.0**	**98.0**	**98.6**	**100**							
10. *L. mesenteroides* ATCC 8293	**95.3**	**95.8**	**95.3**	**95.6**	**95.7**	**98.2**	**98.2**	**98.9**	**99.0**	**100**						
11. *L. mesenteroides* ATCC 8293D-5	**95.3**	**95.8**	**95.3**	**95.6**	**95.7**	**98.2**	**98.2**	**98.9**	**99.0**	**100**	**100**					
12. *L. gelidum* DSM 19374	74.8	74.9	74.7	74.8	74.8	75.4	75.4	74.6	75.0	74.8	74.7	**100**				
13. *L. fallax* ATCC 700006	70.4	70.6	70.5	70.1	70.1	70.6	70.7	70.3	70.3	70.1	70.1	69.7	**100**			
14. *L. falkenbergense* DSM 5624	78.4	78.7	78.8	79.0	79.0	79.4	79.3	79.1	79.6	79.2	79.1	74.8	70.7	**100**		
15. *L. pseudomesenteroides* DSM 20193	78.2	78.4	78.3	78.4	78.4	79.1	79.0	78.3	78.6	78.3	78.3	74.5	70.3	**95.2**	**100**	
16. *L. carnosum* DSM 5576	75.5	75.8	75.5	75.4	75.2	75.7	75.6	75.3	75.6	75.6	75.6	79.0	70.2	75.8	75.2	**100**

*Note: The table shows the average amino acid identity (AAI) percentages among the genomes of *Leuconostoc suionicum* VITA-PB2 and closely related taxa. Each rows and columns represent individual genomes. Values exceeding 95% are shown in bold. *Leuconostoc suionicum* VITA-PB2 highlighted in red.
