# Supplementary material for: Comprehensive genomic and functional analysis of Leuconostoc lactic acid bacteria in alcohol and acetaldehyde metabolism
Source: J Microbiol. 2025 Feb 27;63(2):e2410026. doi: 10.71150/jm.2410026 (PMC13577034; doi:10.71150/jm.2410026)
Supplement: Supplementary file 6 [file jm-2410026-Supplementary-Table-S5.pdf]

Table S5. Genes of interest identified in the VITA-PB2 genome. The locus IDs matched those in Dataset S1.

Metabolism	Gene name	Locus ID
Alcohol metabolism	Alcohol dehydrogenase (adh)	VITAPB2_00023
		VITAPB2_00504
		VITAPB2_01164
		VITAPB2_01882
		VITAPB2_01627
	Aldehyde dehydrogenase (aldh)	VITAPB2_01275
Bacteriocin production	ATP-binding cassette, subfamily C, bacteriocin exporter (lagD)	VITAPB2_01673
		VITAPB2_02115
	Bacitracin transport system ATP-binding protein (bcrA)	VITAPB2_01699
	Lactococcin G-beta/enterocin 1071B family bacteriocin	VITAPB2_02113
	Membrane fusion protein, peptide pheromone/bacteriocin exporter (lcnD)	VITAPB2_01674
		VITAPB2_02116
	Undecaprenyl-diphosphate phosphatase (uppP)	VITAPB2_01640
Electron transport chain (ETC)	Cytochrome bd ubiquinol oxidase subunit I (cydA)	VITAPB2_01205
	Cytochrome bd ubiquinol oxidase subunit II (cydB)	VITAPB2_01204
	Thiol reductant ABC exporter subunit (cydC)	VITAPB2_01202
	Thiol reductant ABC exporter subunit (cydD)	VITAPB2_01203
	NAD(P)/FAD-dependent oxidoreductase	VITAPB2_00837
		VITAPB2_00957
		VITAPB2_01499
	NADH oxidase (nox)	VITAPB2_01739
	NADH peroxidase (npr)	VITAPB2_01653
	NADH:quinone reductase (non-electrogenic) (ndh)	VITAPB2_01568
	NADH-dependent flavin oxidoreductase	VITAPB2_01942

	NADH-dependent oxidoreductase	VITAPB2_01559
Polysaccharides production	Dextransucrase	VITAPB2_00913
		VITAPB2_00914
		VITAPB2_02047
	Levanase (sacC)	VITAPB2_00251
		VITAPB2_00372
		VITAPB2_00562
		VITAPB2_01053
	Levansucrase (sacB)	VITAPB2_00249
		VITAPB2_00250
Tricarboxylic acid (TCA) cycle	Succinate dehydrogenase flavoprotein subunit (frdA)	VITAPB2_01659
